# Possible Mechanisms of Action of Two *Pseudomonas* *fluorescens* Isolates as Probiotics on Saprolegniosis Control in Rainbow Trout (*Oncorhynchus mykiss* Walbaum)

**DOI:** 10.3390/ani10091507

**Published:** 2020-08-26

**Authors:** Concepción González-Palacios, Juan-Miguel Fregeneda-Grandes, José-Miguel Aller-Gancedo

**Affiliations:** Departamento de Sanidad Animal, Universidad de León, Campus de Vegazana s/n, 24071 León, Spain; cgonp@unileon.es (C.G.-P.); jmallg@unileon.es (J.-M.A.-G.)

**Keywords:** biocontrol, saprolegniosis, *Pseudomonas fluorescens*, siderophores production, *Oncorhynchus mykiss*

## Abstract

**Simple Summary:**

In aquaculture, as in other production systems, diseases caused by microorganisms can produce great economic losses and it is essential to have preventive or curative treatments for their control. However, there is increasing concern and restrictions on the use of chemical products for the treatment of such diseases. In this context the use of probiotics could be a good alternative to chemical treatment. Previous studies of our research group have shown the usefulness of two *Pseudomonas fluorescens* isolates for the biocontrol of saprolegniosis in rainbow trout—a disease caused by a pseudo-fungus that affects freshwater fish—under experimental conditions when they are added to the tank water. In the present work we investigated some of the possible mechanisms of action of these two isolates, such as stimulation of the immune response, competition for binding sites or sources of nutrients, and the production of bioactive substances inhibitory to pathogenic agents. To sum up, the two *P. fluorescens* isolates used in the present study might be usable for biocontrol of saprolegniosis and the mode of action of these bacteria is likely to be related to the production of siderophores (small compounds with high affinity binding for iron).

**Abstract:**

Probiotics have been proposed as one of the alternatives to the chemical treatments currently used in aquaculture. Recently, the possible usefulness of certain microorganisms, mainly bacteria, has been highlighted as a potential biocontrol for saprolegniosis. In the present work we investigated the possible mechanisms of action of two isolates of *Pseudomonas fluorescens* (LE89 and LE141) with proven ability to reduce *Saprolegnia parasitica* infection in rainbow trout under experimental conditions when they are added to the tank water. The stimulation of the innate immune response and the production of siderophores and bioactive substances inhibiting *S. parasitica* present in cells and supernatants of LE89 and LE141 were studied. Regarding the immune response the only noteworthy points were the increase in the phagocytic activity of macrophages and the concentration of serum proteins when LE141 was administered. Both bacteria produced siderophores. When analyzing the protein substances present in supernatants, it was observed that in both isolates the proteins with inhibitory activity present might be siderophores. In LE141, besides siderophores, a protein of 66 kDa was identified in the fraction responsible for inhibition. To sum up, the two *P. fluorescens* isolates might be usable for biocontrol of saprolegniosis and that the mode of action of these bacteria is likely to be related to the production of siderophores.

## 1. Introduction

Saprolegniosis produced by *Oomycetes* of the genus *Saprolegnia*, mainly *Saprolegnia parasitica*, is a disease that affects freshwater fish, especially salmonids, at all phases in their biological cycle. On fish farms, saprolegniosis can occur endemically with constant loss of fish and it poses serious problems for incubating eggs. This disease has seen an upwards trend since the prohibition of the use of malachite green, and is thus seen as having a major financial impact on freshwater fish aquaculture [1,2]. Preventive and curative treatments currently employed to control saprolegniosis do not meet all the needs arising in the cultivating of fish and they have a number of drawbacks relating to effectiveness and safety in their use [1].

Probiotics have been proposed as one of the alternatives to the chemical treatments currently used in aquaculture [3,4]. Recently, the possible usefulness of certain microorganisms, mainly bacteria, has been highlighted as a potential biocontrol for saprolegniosis [5,6,7,8,9,10,11].

In previous studies of our research group, various bacterial isolates with an in vitro ability to inhibit the growth of *Saprolegnia parasitica* were obtained [12]. Thereafter, the facts that they were not pathogenic for rainbow trout (*Oncorhynchus mykiss*), and that they could adhere to the cutaneous mucus and reduce the adhesion of zoospores and cysts of *S. parasitica* were all demonstrated [13]. Finally, it was found that of the fifteen isolates investigated, two strains of *Pseudomonas fluorescens*, LE89 and LE141, reduced infection by *S. parasitica* in rainbow trout under experimental conditions when added to the water in the tanks [11].

The aim of the present study was to determine the activity of these two bacteria (LE89 and LE141) for the control of saprolegniosis. It was planned also to investigate some of the mechanisms of action described in probiotics, such as stimulation of the immune response, competition for binding sites and for sources of nutrients and energy, and the production of bioactive substances inhibitory to pathogenic agents [4].

## 2. Materials and Methods 

### 2.1. Stimulation of the Innate Immune Response in Rainbow Trout by Adding LE89 and LE141 to the Water

Rainbow trout (*Oncorhynchus mykiss*) from a commercial fish farm (71.83 ± 9.90 g) was used. The fish were acclimatized for a minimum of 10 days in a 120 L tank during which they were observed daily to check that they did not show clinical signs of disease. Trials were carried out in triplicate with groups of 12 fish, kept in 40 L tanks with chlorine-free well water (renewal rate of 1.5 L per h) at 12 °C. There was constant aeration, and a photoperiod of 12/12 h of light and darkness was used. Effluent water was disinfected with ozone. The experimental protocol was approved by the Subcommittee for Experimentation and Animal Welfare of the University of Leon, Spain (Protocol number ULE_04_2015).

The fish were bathed daily for 14 days by stopping the water flow in the tanks and adding the bacterial suspension at a final concentration of 10^6^ bacteria/mL for 6 h, after which the water flow was resumed. In control tanks the bacterial suspension was replaced with the same volume of sterile phosphate-buffered saline (PBS). Thereafter, four fish from each batch were anaesthetized with tricaine methane sulfonate (MS-222) at a dose of 50 mg/mL. Blood and cutaneous mucus samples were taken by venipuncture of the caudal vein and by scraping the skin surface, respectively. The fish were euthanized by overdosing them with MS-222 (100 mg/mL) and macrophages were extracted from the pronephros.

During the experiments, water temperature was measured daily and other physical–chemical parameters were measured every other day. No differences were observed between the different groups and the values were as follows (mean ± SD): temperature, 12.52 ± 0.16 °C; pH 7.90 ± 0.04; dissolved oxygen, 9.86 ± 0.12 mg/L; nitrites (NO_2_), 0.032 ± 0.005 mg/L; and non-ionized ammonia (NH_3_), 0.059 ± 0.005 mg/L.

In order to determine the stimulation of innate immunity the following parameters were measured: blood cell counts (erythrocytes, leucocytes, and the proportions of lymphocytes, granulocytes, and monocytes), phagocytic capacity and production of superoxide ion by macrophages of the pronephros, total proteins, and lysozyme activity of serum and of the cutaneous mucus.

#### 2.1.1. Blood Cell Counts

Heparinized blood was subjected to three decimal dilutions in sterile PBS (pH 7.2), with the 10^−2^ dilution being used for counting leucocytes and the 10^−3^ dilution for the erythrocyte count. Counting was undertaken in a Neubauer Improved chamber (Assistent, Sondheim vor der Rhön, Germany) at 400 magnifications. In determining the proportions of different types of leucocyte (lymphocytes, granulocytes, and monocytes), samples were placed on a microscope slide and stained using the Diff-Quick procedure, 100 leucocytes being counted in triplicate [14].

#### 2.1.2. Phagocytic Capacity and Superoxide Ion Production by Macrophages of the Pronephros

Macrophages were obtained using the method previously described [15]. In brief, the head kidney was extracted aseptically and broken up with scissors into maintenance medium (RPMI 1640 medium without phenol red, 2% fetal bovine serum-FBS, 50 µg/mL gentamicin, and 2 µg/mL amphotericin) supplemented with 20 IU of heparin. The pronephros homogenate was passed through a 100 µm nylon mesh and the macrophages were collected from the interphase of a discontinuous gradient of Percoll^®^ (GE Healthcare Life Science, Uppsala, Sweden) 34:51% (v:v) in Hank’s balanced salt solution-HBSS, after centrifuging at 400× *g* for 25 min at 4 °C. The macrophages were rinsed twice with RPMI 1640 (400× *g* for 5 min at 4 °C), then resuspended in maintenance medium without heparin. The number of viable cells was checked by adding trypan blue (0.1%), then counting in a Neubauer chamber and finally adjusting the concentration to 10^7^ cells/mL.

Phagocytic activity was measured according to Mathews et al. [16] with slight modifications. Into a sterile polystyrene tube, 1 mL of the macrophages suspension and 1 mL of a total of 10^9^ latex spheres 0.85 µm in diameter (Sigma-Aldrich^®^, St. Louis, MO, USA) resuspended in RPMI 1640 were added. Tubes were incubated at 20 °C for 1 h with 5% CO_2_. After incubation, 1 mL of freshly thawed sterile PBS was added, and the mix was centrifuged at 275× *g* for 5 min. The pellet was then laid on a microscope slide, stained with Diff-Quick and a count of 100 cells was carried out in triplicate to determine the percentage of macrophages phagocytizing latex spheres relative to the total number of macrophages.

Superoxide ion production was made in a 96-well microplate, 100 µL of the macrophages suspension was placed in maintenance medium supplemented with 0.1% FBS. Plates were incubated for 2 h at 20 °C with 5% CO_2_. After incubation, the wells were rinsed twice with RPMI 1640 to remove non-adhered cells. Thereafter, each well had added to it 100 µL of RPMI supplemented with 1 mg/mL of nitro blue tetrazolium (NBT) and 1 µg/mL of phorbol 12-myristate 13-acetate (PMA) dissolved in dimethyl sulfoxide (DMSO). Plates were incubated for 1 h at 20 °C, the supernatant was then eliminated and the reduction of NBT was halted by adding absolute methanol. The resulting formazan was dissolved by adding to the wells 120 µL of KOH 2M and 140 µL of DMSO. Plates were read by means of spectrophotometry at 620 nm every 10 min for 1 h using KOH and DMSO as blanks [17].

#### 2.1.3. Lysozyme Activity in Serum and Cutaneous Mucus

The protocol described by Lange et al. [18] was used: 100 µL of serum and four successive double dilutions of this (1:5 to 1:40) were made in alkaline phosphate buffer. These were deposited in a 96-well plate and 100 µL of a suspension of 0.4 mg/mL of *Micrococcus luteous* (*lysodeikticus*) (Sigma-Aldrich^®^, St. Louis, MO, USA) was added. As a positive control, the serum was replaced with 1.6 µg/mL of egg-white lysozyme and four double dilutions of this in phosphate buffer. As a negative control, the serum was replaced with the same volume of phosphate buffer. After incubation of the plates—1 h at 20 °C—the optical density at 570 nm was measured every 15 min for 1 h and lysozyme units were established (the quantity of serum able to decrease the optical density of the sample by 0.001 per min). Cutaneous mucus was obtained by scraping the surface of the fish, then homogenized with sterile PBS and centrifuged at 27,000× *g* for 15 min at 4 °C to remove cell debris and particles. To determine lysozyme activity, the mucus was prepared following the method in Ellis [19], it was homogenized with four parts (w/v) of PBS 0.004 M and centrifuged at 10,000× *g* for 10 min. The supernatant for determining lysozyme activity was obtained by using the same protocol as that employed for blood serum.

#### 2.1.4. Total Proteins in Serum and Cutaneous Mucus

These were determined by spectrophotometry, measuring absorbance at 280 nm with a NanoDrop ND-1000 (Thermo Fisher Scientific, Wilmington, DE, USA). As controls, samples of bovine serum albumin of a known concentration were used.

#### 2.1.5. Statistical Analysis

The data obtained were compared using the non-parametric Kruskal–Wallis test for the data in which the median and ranges were obtained. Continuous data and percentages were compared through one-way analysis of variance (ANOVA), percentages first being subjected to arc-sine transformation [20]. Statistical analysis was carried out with the Epi Info™ 7 software for Windows, version 7.2.1.0, (Centers for Disease Control and Prevention, CDC, Atlanta, GA, USA) taken *p* < 0.05 as the level of significance.

### 2.2. Production of Siderophores

*Pseudomonas fluorescens* LE89 and LE141 were inoculated into Chrome Azurol S/hexadecyltrimethylammonium bromide (CAS-HDTMA) culture medium at 20 °C. After 24 and 48 h checks were made on the appearance of a color change in the medium caused by the reduction of ferric ion (Fe^+3^) by the siderophores produced by the bacteria [21].

CAS-HDTMA medium was prepared by mixing 5 mL of CAS solution (12.1 mg/mL of chrome azurol S in distilled water) with 1 mL of a solution of iron chloride (0.2703 mg FeCl_3_ 6H_2_O, 0.001 mL HCl 1N and 0.99 mL distilled water) and 4 mL of a solution of HDTMA (1.8225 mg/mL of hexadecyltrimethylammonium bromide in distilled water). This mixture was added to 90 mL of tryptone soy agar (TSA) and was sterilized by heating to 121 °C for 15 min. The medium was allowed to stand, and then 100 µL of a 0.1 M solution of 2,2’-dipyridyl was added before it was dispensed onto Petri dishes.

### 2.3. Substances Inhibiting Saprolegnia Parasitica Present in Cells and Supernatants of LE89 and LE141

To determine the type of substance responsible for the inhibition, tests were carried out of the inhibition of growth of *S. parasitica* in a liquid medium, using a colonized hemp seed, and of germination of cysts. These were undertaken with bacterial cells and supernatants from cultures subjected to various heat treatments, and also with the cytoplasmic substances and cell debris of the bacteria.

To obtain bacterial cells, strains LE89 and LE141 were cultured overnight at 20 °C in 3 mL of TSB (Tryptone Soy Broth); 500 µL of this suspension was transferred to 250 mL of TSB and incubated at 20 °C for 24 h with constant agitation at 200 rpm until the exponential growth phase. Thereafter, the culture was centrifuged at 1000× *g* for 15 min and the pellet was resuspended in sterile physiological serum, the concentration being adjusted to 2 × 10^5^ bacteria/mL by reading the optical density at 540 nm (for the inhibition of the germination of cysts 4 × 10^5^ bacteria/mL were used). From the initial concentration, five serial decimal dilutions were performed in sterile physiological serum with three aliquots of each solution being taken. One of these was given no heat treatment, another was treated at 70 °C for 1 h and the third at 100 °C for 5 min.

To obtain the supernatants LE89 and LE141 were incubated in 3 mL of TSB at 20 °C overnight, and an aliquot of 250 µL was inoculated into 200 mL of tryptone-neutralized peptone broth (TNPB) and incubated at 25 °C for 36 h with constant agitation at 200 rpm [22]. This culture was centrifuged at 15,000× *g* for 20 min. The supernatant obtained was passed through a 0.22 µm filter, and from this initial supernatant three double dilutions were made in TNPB. Three aliquots of each were taken and the same heat treatments as indicated for bacterial cells were performed.

Cytoplasmic substances and cell debris were obtained by breaking down bacterial cells with 0.17 mm glass beads and a Fast-Prep24^TM^ homogenizer (MP Biomedicals, Solon, Ohio, USA) with 10 pulses of 45 s. After the cells were broken down, tubes were centrifuged at 1000× *g* for 10 min. This separated the cytoplasmic content present in the supernatant from the cell debris contained in the pellet. These latter were resuspended in 1 mL of sterile physiological serum.

For the inhibition test of mycelium growth of *S. parasitica*, 1 mL of each bacterial dilution (heat treatment or not), 1 mL of TSB and a half hemp seed colonized by *S. parasitica* were dispensed by duplicate into each well of a 24-well tissue culture plate (Falcon). In the case of supernatants, cytoplasmic content or cellular debris, 2 mL of these and no TSB were added. Test for inhibition of cyst germination were carried out basically like the inhibition of mycelium growth, the only difference being that 0.5 mL of a zoospore suspension of *S. parasitica* (4 × 10^4^ zoospores/mL) was added in place of a colonized hemp seed, and 0.5 mL of each bacterial dilution was used instead of 1 mL. Bacteria and *S. parasitica* negative controls were included on each plate. Plates were incubated for 3 d at 20 °C. The presence/absence of macroscopic or microscopic hyphal growth and the germination of cysts were observed throughout the incubation period using a Nikon Diaphot inverted microscope and results were recorded on Day 3.

To study the protein-compounds present in the supernatants, the method previously described [22] was used, but with two different culture mediums, TNPB, as employed by those authors, and TSB. The protein compounds in the supernatants were precipitated with ammonium sulfate at an initial saturation of 80% and thereafter with sequential precipitations at 30%, 45%, 60%, and 80% [23]. The precipitated fractions were centrifuged at 14,000× *g* for 20 min. They were resuspended in 2 ml of PBS and desalinated by passing them through a PD-10 Desalting Column (GE Healthcare Life Science, Uppsala, Sweden) [24]. Each eluate was checked for its ability to inhibit the development of the mycelium and the germination of cysts of *S. parasitica*. In those which proved inhibitory, the total protein concentration was determined with a NanoDrop^®^ ND-100 (Thermo Fisher Scientific, Wilmington, DE, USA) and checked whether or not the protein fraction presented siderophores by adding 1 mL of the eluate to 1 mL of CAS culture medium in which TSA medium was replaced with TSB. The number of proteins present in the eluates and their molecular weight were estimated by electrophoresis in polyacrylamide gel with sodium dodecyl sulfate (SDS) at 200 V for 75 min. The gel obtained was stained with Coomassie brilliant blue and the molecular weight of the proteins was estimated by using a low molecular weight sodium dodecyl sulfate (LMW-SDS) marker from GE Healthcare Life Science (Uppsala, Sweden).

## 3. Results

### 3.1. Stimulation of the Innate Immune Response in Rainbow Trout by Adding LE89 and LE141 to the Water

There were no statistically significant differences in the counts of erythrocytes and leucocytes between treated fish and their controls, or within different leucocytes subtypes (Table 1 and Table 2).

With regard to serum lysozyme activity, the differences with respect to controls were significant only with LE141 (H = 9.01; *p* = 0.003), but levels were higher in control fish than in those treated with the bacteria. In respect of lysozyme activity of the cutaneous mucus, neither of the two bacteria showed significant differences relative to controls, although their values in both cases were higher than those in treated fish, as had occurred with serum lysozyme (Table 1 and Table 2).

In the case of total protein concentration in serum, in both cases significant differences were observed, but with LE89, control fish presented higher concentrations than treated (H = 3.88; *p* = 0.049), while in contrast with LE141 there was a significant increase in treated fish relative to controls (H = 8.37; *p* = 0.004). No significant differences were observed in the concentration of proteins present in the cutaneous mucus (Table 1 and Table 2).

**Figure 1:**
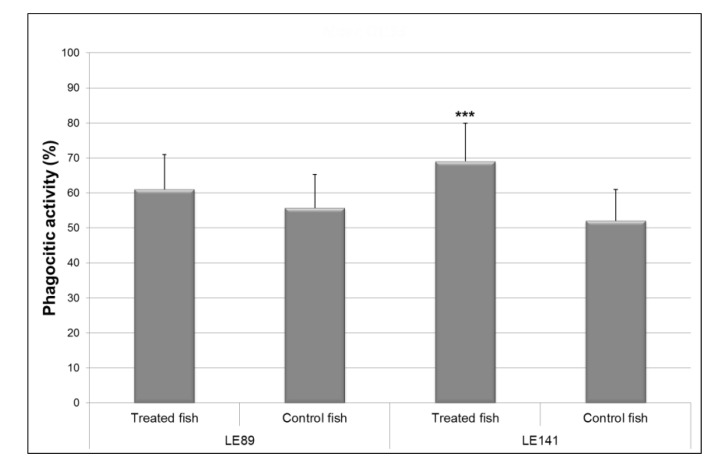
Phagocytic activity of the macrophages of the pronephros in rainbow trout treated with *Pseudomonas fluorescens* LE89 or LE141 in the tank water. Data are represented as mean ± standard deviation of 12 fish (4 fish from each of the three experimental replicas); *** Statistically significant difference (*p* ≤ 0.001) with respect to controls.

Treatment with both bacteria increased phagocytic activity of macrophages relative to controls. However, differences were significant (F = 15.74; *p* < 0.001) only in the case of LE141 (Figure 1).

**Figure 2:**
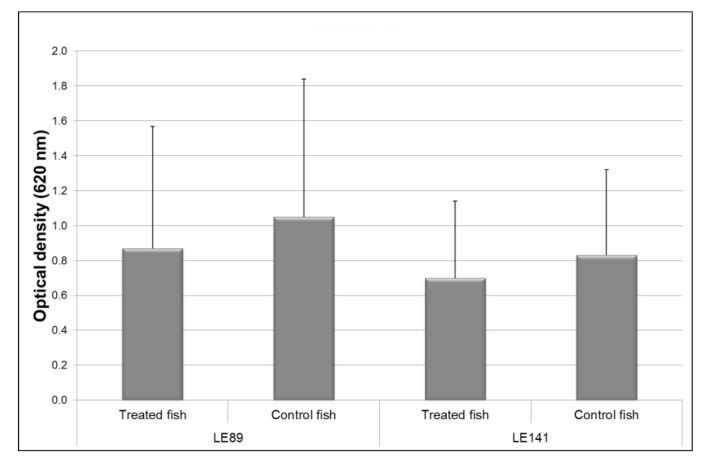
Superoxide ion production by macrophages of the pronephros in rainbow trout treated with *Pseudomonas fluorescens* LE89 or LE141 in tank water. Data are represented as mean ± standard deviation of 12 fish (4 fish from each of the three experimental replicas).

Superoxide ion production by macrophages of the fish treated with LE89 and LE141 bacteria was less than in controls. However, the differences were not significant (Figure 2).

### 3.2. Production of Siderophores

**Figure 3:**
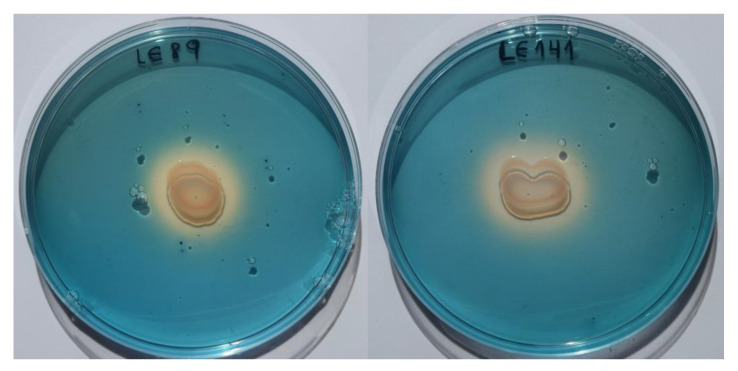
Production of siderophores by *Pseudomonas fluorescens* LE89 (**left**) and LE141 (**right**) in the CAS-HDTMA medium.

Cultures on CAS-HDTMA medium demonstrated that both LE89 and LE141 produced siderophores. With both bacteria a change was observed in the coloring of the medium, from blue to yellow, during the first 24 h of incubation (Figure 3).

### 3.3. Substances Inhibiting Saprolegnia Parasitica Present in Cells and Supernatants of LE89 and LE141.

Live cells of LE89 and LE141 without heat treatment produced a high level of inhibition of both mycelial growth and cyst germination of *S. parasitica*. When the cells were heat treated at 70 °C for 1 h or 100 °C for 5 min, none of the bacteria inhibited the growth of the mycelium or cysts germination. Similarly, supernatants of cultures showed an inhibitory effect which disappeared when they underwent heat treatment. The inhibitory effect was greater in the case of cells (total absence of growth with the first dilution) than with supernatants (partial inhibition with the first dilution). In contrast, cell debris and cytoplasmic content did not inhibit the growth of *S. parasitica*, whether or not they were subjected to heat treatment (Table 3 and Table 4).

**Figure 4:**
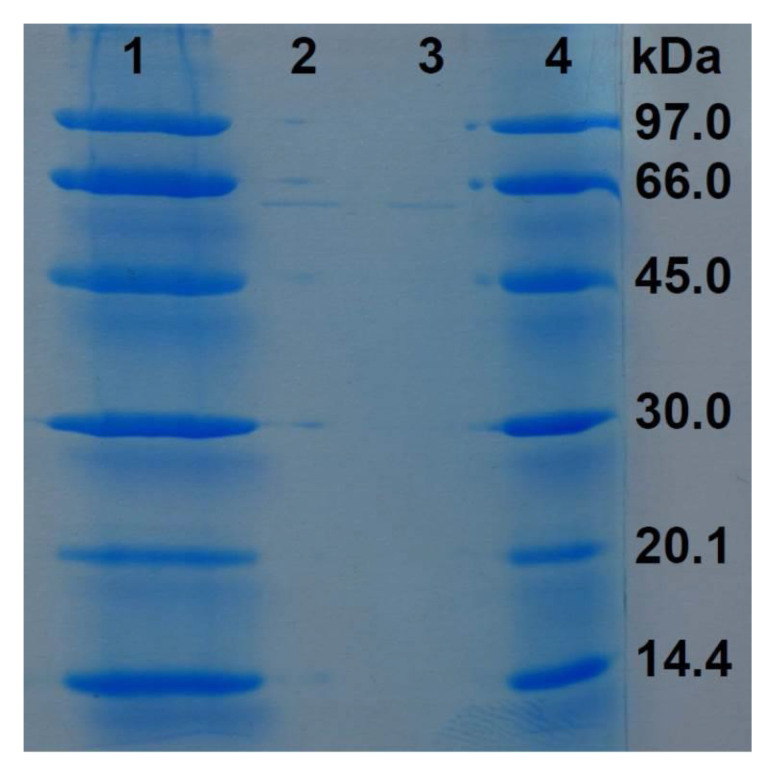
Detection of a peptide presents in the 45–60% protein precipitation fraction of the *Pseudomonas fluorescens* LE141 supernatant (SDS-PAGE stained with Coomassie brilliant blue; lanes 1 and 4 are the LMW marker from GE Healthcare Life Science (Uppsala, Sweden); lanes 2 and 3 are the purified peptide).

**Figure 5:**
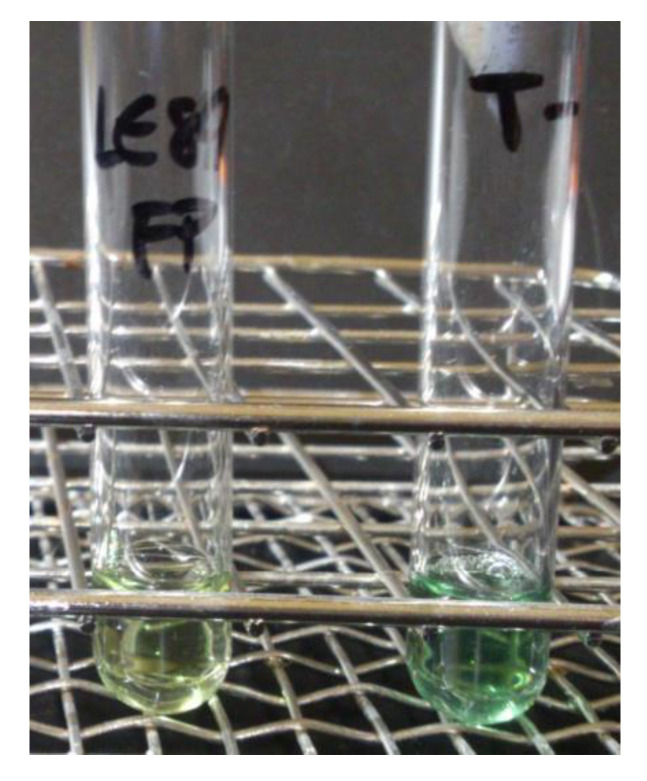
Presence of siderophores in the 30% protein precipitation fraction of the *Pseudomonas fluorescens* LE89 supernatant perceived by the color change in CAS (Chrome Azurol S/hexadecyltrimethylammonium bromide) broth medium (on the left side) versus a negative control with only PBS (phosphate-buffered saline) (on the right side).

The eluates of the precipitation with an 80% saturation of ammonium sulfate of the proteinaceous components of supernatant of LE89 and LE141 inhibited both development of the mycelium and germination of cysts. This also occurred with the protein fraction obtained through precipitation with 30% ammonium sulfate, and in the case of LE141 when fractions precipitated between 45% and 60% were employed. In electrophoresis of the 45% to 60% fraction of LE141 a peptide band was observed with an approximate molecular weight of 66 kDa (Figure 4). In the 30% fraction of both isolates no peptide was observed in electrophoresis. However, when the eluate was incorporated into CAS broth medium, a change in color occurred after just a few minutes, which indicated the presence of siderophores in this fraction (Figure 5).

## 4. Discussion

In a previous work of our research group [11] the addition of two isolates of *Pseudomonas fluorescens*, LE89 and LE141, to the water in tanks for 14 days at a concentration of 10^6^ bacteria/mL was able to reduce experimental infection with *Saprolegnia parasitica* in rainbow trout. In fact, fish treated with LE89 had a 24.5% infection rate, and those treated with LE141 a rate of 42.8%, while controls showed 100% of infection. The aim of the present study was to investigate the possible modes of action of these bacteria in protecting against saprolegniosis.

Among the various mechanisms of action described for probiotics, modulation of the immune system is one of the most commonly purported benefits [25]. However, stimulation of the innate immune response by the two strains of *P. fluorescens* used in this work, LE89 and LE141, seems unlikely. The only noteworthy points were the increase in the phagocytic capacity of macrophages and the concentration of serum proteins when LE141 was administered. Additionally, lysozyme activity both of serum and of cutaneous mucus, and respiratory burst of macrophages in treated fish was smaller than in controls, and these differences were significant in some instances. In the case of LE89 isolate the only parameter that presents differences with the controls was serum protein concentration but it was lower in the treated group, however it must be taken into account that the level of significance (*p* = 0.049) is very close to the level of cut off value (*p* = 0.05).

Nevertheless, stimulation of the phagocytic activity of macrophages in the case of LE141 might indicate a certain effect. In fact, an immune response of a cellular type in channel catfish (*Ictalurus punctatus*) that was effective against *S. parasitica* has been described [26].

Our results differ from the findings of Eissa et al. [27] who supplemented the diet with *Pseudomonas fluorescens* in Nile tilapia (*Oreochromis niloticus*) and observed a significant reduction in the mortality rate and an increase in hematological parameters, total protein, and globulin after challenge with *Pseudomonas anguilliseptica* and *Streptococcus faecium*. However, our results do coincide with the findings by Korkea-aho et al. [28] who saw no rise in blood cell counts, phagocytic activity or serum and intestinal mucus lysozyme activity in rainbow trout after administration of *Pseudomonas* sp. M174 in the feed for two weeks. However, the respiratory burst activity of the head kidney macrophages was higher in M174 fed fish than in the controls. This was despite the fact that this strain has been demonstrated to prevent flavobacteriosis. In this study, some of the parameters analyzed were also lower in the treated fish than in the controls, as in our work.

The mechanisms by which probiotics stimulate the immune system are not yet well understood, but it is known that factors such as type of strain, dose, duration, and mode of administration or environmental conditions can affect the immunomodulating potency of probiotics [25]. In this work, a dose of 10^6^ bacteria/mL in the water tank during two weeks was used, which may not have been sufficient to stimulate the immune system, but has been found to be effective in vivo to reduce experimental infection with *S. parasitica* [11].It has been suggested that immune cells in hosts do not react against the bacteria that are naturally found on their surfaces as strongly as they do against bacteria normally not present in their surroundings [29]. This might explain the very limited stimulation of the immune response by LE89 and LE141, as they were isolated from the skin surface of brown trout (*Salmo trutta*) and rainbow trout (*Oncorhynchus mykiss*) respectively [12].

Another of the modes of action described in probiotics is the competitive exclusion due to the occupation of binding sites for pathogens, or the probiotic uptake of essential nutrients like iron [4]. The probiotic action of LE89 and LE141 by means of competitive exclusion seems to be well grounded, as both isolates produced siderophores and in a previous work [13] demonstrated its ability to adhere to the cutaneous mucus of trout, impeding the fixation of *S. parasitica* cysts.

Various authors have described as a mechanism of action of the genus *Pseudomonas* the competition with other microorganisms by means of iron reduction through the production of siderophores [28,30,31,32]. The presence of two different types of siderophores in *Pseudomonas fluorescens* ATCC 17400 has been demonstrated [33]; these were a pyoverdine and a quinolobactin associated with the antifungal effect of this bacterium against an oomycete of the genus *Pythium*. Furthermore, the case of the in vitro inhibitory ability of *P. fluorescens* against *S. parasitica* has been associated with the production of siderophores [34,35]. However, they also suggested the possibility that these isolates might produce some substances with an enzymatic or antibiotic activity responsible for the inhibitory effect.

A number of studies have described the production by *Pseudomonas* spp. of a range of substances of varying nature and effects [36,37,38,39]. Thus, the presence of a surfactant lipopeptide produced by *Pseudomonas* sp. isolate H6 inhibited growth of *Saprolegnia* spp. in vitro but no significant protection of salmon eggs against saprolegniosis was observed in vivo, while the addition of live inoculum of the isolate H6 significantly reduced the mortality in salmon eggs caused by *Saprolegnia diclina* [7]. It has also been suggested [9] that the effectiveness of various bacterial isolates in reducing infection by *Saprolegnia* sp. in eggs of the giant gourami (*Osphonemus goramy*) would be due to their ability to adhere to the surface of the eggs and the secretion of exoenzymes with chitinase and glucanase activity.

In the present study, both cells and supernatants of LE89 and LE141 not receiving any heat treatment did present an inhibitory effect both on mycelial growth and cysts germination of *S. parasitica*. This inhibitory effect disappeared when any heat treatment was applied (whether at 70 °C for 1 h or at 100 °C for 5 min) which suggests the proteinaceous nature of the inhibitory substances. When analyzing the protein substances present in supernatants, it was observed that in both isolates the proteins with inhibitory activity present might be siderophores. In LE141, besides siderophores, a protein of 66 kDa was identified in the fraction responsible for inhibition. These results would be coincident with the findings of Wang and Zhang [23], who isolated a peptide of molecular weight 58 kDa in the supernatant of cell rupture of *Pseudomonas protegens* XL03 that was capable of in vitro inhibition of cysts germination of *S. parasitica*, with its activity disappearing at temperatures above 50 °C.

Apart from various species of *Pseudomonas*, the usefulness of other microorganisms for biocontrol of saprolegniosis has also been investigated. Thus, an *Aeromonas media* A199 isolate was successfully used to prevent saprolegniosis in silver perch (*Bidyanus bidyanus*) and in the short-finned eel (*Anguilla australis*) [5,6]. In subsequent studies, it was determined that the substance responsible for inhibition was the indole produced by the bacterium [22]. In another study [10] the application of supernatant from a culture of a bacterium identified as *Burkholderia* sp. produced a reduction of 53.3% in the rate of infection by *Saprolegnia* sp. in grass carp (*Ctenopharyngodon idella*). The substance with antifungal activity detected in the supernatant was thermostable and was identified as 2-pyrrolidone-5-carboxylic acid, a derivate of glutamic acid.

## 5. Conclusions

To sum up, the two isolates of *P. fluorescens*, LE89 and LE141, obtained from the skin of brown trout and rainbow trout, respectively, might be usable for the biocontrol of saprolegniosis caused by *S. parasitica* in rainbow trout by adding them to the water. The mode of action of these bacteria is likely to be related to competitive exclusion from binding sites on the skin surface, to the production of siderophores, and to the generation of extracellular substances of a proteinaceous nature. In contrast, stimulation of the innate immune response does not appear to play any relevant part. Furthermore, it is not possible to rule out a combination of various different mechanisms of action.

Pseudomonads are common components of the microbiota of fish and freshwater ecosystems and have been widely studied for biocontrol purposes in aquaculture, mainly the species *P. fluorescens* [7,27,28,30,31,32,34,35]. Some isolates of *P. fluorescens* have been considered as fish spoilage or secondary invaders of damaged fish tissues but are only rarely reported as primary pathogens of fish [40]. However, it may become necessary to assess whether the two *P. fluorescens* isolates used in the present study that were not pathogenic for rainbow trout [13] would be equally benign against other fish species, particularly other salmonids, if they were intended to be used for biological control of saprolegniosis.

## Figures and Tables

**Table 1:** Parameters of the innate immune response in rainbow trout when *Pseudomonas fluorescens* LE89 is administered to the water.

Parameter (Unit)	Treated Fish ^1^	Control Fish ^1^
Erythrocytes (× 10^9^ cells/mL)	4.96 (3.83:7.00)	4.96 (1.75:7.67)
Leucocytes (× 10^8^ cells/mL)	1.79 (0.67:2.83)	1.29 (0.58:2.42)
Lymphocytes (%)	92 (64:100)	84 (67:100)
Granulocytes (%)	8 (0:32)	12 (0:33)
Monocytes (%)	0 (0:8)	0 (0:8)
Serum lysozyme activity (U/mL)	7.78 (6.12:12.98)	9.79 (4.30:17.84)
Cutaneous mucus lysozyme activity (U/mL)	10.96 (8.10:30.12)	13.17 (7.59:19.71)
Serum protein concentration (mg/mL)	35.53 (25.63:43.42) *	38.79 (35.56:44.28)
Cutaneous protein concentration (mg/mL)	40.79 (18.54:86.40)	43.67 (8.52:96.08)

^1^ Results expressed as median (minimum:maximum) of 12 fish (4 fish from each of the three experimental replicas); * Statistically significant difference (*p* ≤ 0.05) with respect to controls.

**Table 2:** Parameters of the innate immune response in rainbow trout when *Pseudomonas fluorescens* LE141 is administered to the water.

Parameter (Unit)	Treated Fish ^1^	Control Fish ^1^
Erythrocytes (× 10^9^ cells/mL)	3.00 (1.42:4.67)	3.41 (1.25:6.42)
Leucocytes (× 10^8^ cells/mL)	1.13 (0.5:2.92)	1.62 (0.17:2.42)
Lymphocytes (%)	84 (64:96)	80 (64:92)
Granulocytes (%)	12 (0:28)	16 (8:32)
Monocytes (%)	8 (0:12)	2 (0:8)
Serum lysozyme activity (U/mL)	9.75 (5.56:15.50) *	14.08 (9.70:29.51)
Cutaneous mucus lysozyme activity (U/mL)	51.06 (16.19:65.31)	59.05 (23.47:80.14)
Serum protein concentration (mg/mL)	37.21 (31.95:42.99) *	31.73 (9.60:37.31)
Cutaneous protein concentration (mg/mL)	47.18 (29.36:111.40)	50.22(35.36:130.94)

^1^ Results expressed as median (minimum:maximum) of 12 fish (4 fish from each of the three experimental replicas); * Statistically significant difference (*p* ≤ 0.05) with respect to controls.

**Table 3:** Inhibitory activity of the cells, supernatant, cell debris and cytoplasmic content of *Psudomonas fluorescens* LE89 (without heat treatment, treatment at 70 °C–1 h or treatment at 100 °C–5 min) on mycelial growth and cysts germination of *Saprolegnia parasitica*.

Component	Treatment	Mycelial Growth	Cyst Germination
	No	High (20) ^1^	High (20)
Cells	70 °C–1 h	No effect	No effect
	100 °C–5 min	No effect	No effect
	No	Low ^2^	Low
Supernatant	70 °C–1 h	No effect	No effect
	100 °C–5 min	No effect	No effect
	No	No effect	No effect
Cell debris	70 °C–1 h	No effect	No effect
	100 °C–5 min	No effect	No effect
	No	No effect	No effect
Cytoplasmic content	70 °C–1 h	No effect	No effect
	100 °C–5 min	No effect	No effect

^1^ High inhibition indicates total absence of growth in the first dilution (minimum number of cells that produce inhibition); ^2^ Low inhibition indicates partial growth in the first dilution.

**Table 4:** Inhibitory activity of the cells, supernatant, cell debris, and cytoplasmic content of *Psudomonas fluorescens* LE141 (without heat treatment, treatment at 70 °C–1 h or treatment at 100 °C– 5 min) on mycelial growth and cysts germination of *Saprolegnia parasitica*.

Component	Treatment	Mycelial Growth	Cyst Germination
	No	High (20) ^1^	High (20)
Cells	70 °C–1 h	No effect	No effect
	100 °C–5 min	No effect	No effect
	No	Low ^2^	Low
Supernatant	70 °C–1 h	No effect	No effect
	100 °C–5 min	No effect	No effect
	No	No effect	No effect
Cell debris	70 °C–1 h	No effect	No effect
	100 °C–5 min	No effect	No effect
	No	No effect	No effect
Cytoplasmic content	70 °C–1 h	No effect	No effect
	100 °C–5 min	No effect	No effect

^1^ High inhibition indicates total absence of growth in the first dilution (minimum number of cells that produce inhibition); ^2^ Low inhibition indicates partial growth in the first dilution.
